# Nucleolar and spindle associated protein 1 promotes metastasis of cervical carcinoma cells by activating Wnt/β-catenin signaling

**DOI:** 10.1186/s13046-019-1037-y

**Published:** 2019-01-24

**Authors:** Han Li, Weijing Zhang, Ming Yan, Jiaqi Qiu, Jueming Chen, Xiaoying Sun, Xiangfu Chen, Libing Song, Yanna Zhang

**Affiliations:** 1 State Key Laboratory of Oncology in South China; Collaborative Innovation Center for Cancer Medicine, Sun Yat-sen University Cancer Center, Guangzhou, 510060 China; 2 Department of Obstetrics Gynecology, The First Pepole’s Hospital, Foshan, Guangdong China

**Keywords:** NUSAP1, Cervical cancer, Metastasis, Epithelial-mesenchyme transition, Cancer stem cell, Wnt/ β-catenin signaling

## Abstract

**Background:**

The primary obstacle to treat cervical cancer is its high prevalence of metastasis, which severely affects patients’ quality of life and survival time. Nucleolar and spindle associated protein 1 (NUSAP1) has been implicated in the development, progression, and metastasis in several types of cancer. However, its oncogenic role in cervical cancer remains unclear.

**Methods:**

Western blot assay and immunohistochemistry were used to determine the expression of NUSAP1 in 21 clinical fresh Cervical cancer tissues and 233 clinicopathologically characterized cervical cancer specimens. The biological roles of NUSAP1 in the metastasis of cervical cancer were investigated both in vitro by EMT, Side population analysis and Transwell assays and so on, and in vivo using a mouse 4w model of hematogenous metastasis and lymph node metastasis. Bioinformatics analysis, luciferase reporter analysis, immunoprecipitation and immunoblotting of nuclear and cytoplasmic cellular fractions were applied to discern and examine the relationshipbetween NUSAP1 and its potential targets.

**Results:**

The results demonstrated that NUSAP1 was upregulated in cervical cancer cells and tissues, correlated positively with metastasis and poor clinical outcome of patients. High expression of NUSAP1 promoted metastasis by enhancing cancer stem cell (CSC) traits and epithelial-mesenchyme transition (EMT) progression, while silencing of *NUSAP1* reduced CSC traits and EMT progression. Mechanistically, upregulation of NUSAP1 induced SUMOylation of TCF4 via interacting with SUMO E3 ligase Ran-binding protein 2 (RanBP2) and hyperactivated Wnt/β-catenin signaling in cervical cancer cells. Additionally, NUSAP1-induced cervical cancer cells metastasis and the cancer stem cell phenotype were abrogated with the Wnt/β-catenin signaling inhibitor XAV-939 treatment. Importantly, co-therapy of conventional treatment and XAV-939 will provide a novel and effective treatment for NUSAP1-ovexpressed cervical cancer patients.

**Conclusions:**

Our results demonstrate thatNUSAP1 upregulation contributes to metastasis of cervical cancer by promoting CSC properties and EMT via Wnt/β-catenin signaling and XAV-939 might serve as a potential tailored therapeutic option for patients with NUSAP1-ovexpressed cervical cancer.

**Electronic supplementary material:**

The online version of this article (10.1186/s13046-019-1037-y) contains supplementary material, which is available to authorized users.

## Background

Cervical cancer is the fourth leading cause of tumor-related death in women worldwide, with an incidence of 530,000 new cases annually [1]. With recent advances in surgery, chemotherapy, and radiotherapy, the 5-year survival rate of patients with cervical carcinoma undergoing standardized treatment can be up to 80%. However, once local metastasis (especially lymph node metastasis) or distant metastasis occurs, the 5-year survival rate drops to barely 50% [2]. The development of metastasis is a serious limitation of long-lasting, effective treatments for cervical cancer patients [3, 4]. Thus, there is an urgent need to explore the underlying molecular mechanism of cervical carcinoma metastasis and to identify new therapeutic targets.

Nucleolar and spindle associated protein 1 (NUSAP1) binds microtubules, and functions in mitotic progression, spindle formation, and stability [5, 6]. Previous studies showed that NUSAP1 is upregulated in several cancers, including prostate cancer, colorectal cancer, and astrocytoma [7–10]. High expression of NUSAP1 was markedly associated with unfavorable prognosis of breast cancer and melanoma [11, 12]. Moreover, overexpression of NUSAP1 enhanced the progression and invasion of astrocytoma and prostate cancer cells [9, 10]. These observations suggested that upregulation of NUSAP1 correlates with cancer progression. However, the function of NUSAP1 in cervical cancer remains unknown.

The Wnt/β-catenin signaling pathway has a pivotal role in regulating metastatic ability, and is constitutively activated in numerous cancers, including cervical cancer [13–17]. Upon activation, Wnt ligands bind to its frizzled receptors and lipoprovein receptor-related protein-5 or − 6 (LRP5/6) co-receptors, which induce translocation and accumulation of β-catenin in the nucleus. Next, β-catenin interacts with the T-cell factor/lymphoid enhancer factor (TCF/LEF) transcription factor to form a complexin cell nuclei, which activates the transcription of target genes. Moreover, the Wnt/β-catenin pathway has a prominent role in the progression processes of various cancers, including epithelial mesenchymal transition (EMT) and cancer stem cell (CSC) properties, which contribute to metastasis in multiple types of cancer [18–23]. However, the association between metastasis and the Wnt/β-catenin pathway in cervical carcinoma remains unclear. Therefore, the present study analyzed the role of NUSAP1 in cervical cancer, and we assessed the association with the Wnt/β-catenin pathway in the progression and metastasis of cervical cancer.

SUMOylation is a type of post –translational modification that a target protein is covalently conjugated with the small ubiquitin-like modifier (SUMO) to specific lysine residues through formation of isopeptide bonds [24, 25]. SUMOylation is a multi-step reaction that is sequentially catalyzed by a SUMO-activating E1 enzyme, the single conjugating E2 enzyme Ubc9, and an E3 ligase, such as members of the PIAS/Siz protein family or RanBP2, which act in concert with Ubc9 as enhancers in the modification process. SUMO modification generally alters the properties of the modified target by influencing –either positively or negatively –its interactions with other cellular factors [26]. Additionally, SUMOylation has attracted increasing attention as widely involved in carcinogenesis, cancer cell proliferation, metastasis and apoptosis, implicating SUMOylation as an important regulator of the progression of cancer [27]. However, the roles of SUMOylation in the aspects of cervical cancer cells development and progression, especially in metastasis and invasion, remain unclear.

The results showed that NUSAP1 was upregulated in cervical cancer cell lines, and NUSAP1 could serve as an independent prognostic indicator for unfavorable clinical outcomes. Moreover, NUSAP1 induced SUMOylation of TCF4 via interacting with SUMO E3 ligase Ran-binding protein 2 (RanBP2) and potently activated the Wnt/β-catenin signaling pathway, thus contributing to the metastatic and CSC-likeproperties of cervical cancer cells. Therefore, our study revealed a potential mechanism for Wnt/β-catenin pathway activation, and suggested NUSAP1 as a novel therapeutic target in patients with cervical carcinoma.

## Methods

### Cell culture

Human cervical cancer cells HeLa, CaSki, C33A, SiHa, MS751, and ME-180,and the human cervical immortalized cell line ECT1/E6E7, were purchase from the American Type Culture Collection (ATCC, Manassas, VA, USA). HCC94 and HeLa229 cell lines were obtained from Shanghai Chinese Academy of Sciences cell bank (China). HeLa, CaSki, SiHa and MS751 cells were grown in Eagle’s Minimum Essential Medium (ATCC-30-2003) supplemented with 10% fetal bovine serum (FBS, Life Technologies). HCC94 cells were grown in RPMI-1640 medium (Life Technologies, Carlsbad, CA, US) supplemented with penicillin G (100 U/ml), streptomycin (100 mg/ml) and 10% fetal bovine serum (FBS, Life Technologies). HeLa229 and ME-180 cells were grown in Dulbecco’s modified Eagle’s medium (Invitrogen) supplemented with 10% FBS.ECT1/E6E7 cells were cultured in Keratinocyte-Serum Free medium (GIBCO-BRL 17005–042) with 0.1 ng/ml human recombinant EGF, 0.05 mg/ml bovine pituitary extract, and additional calcium chloride 44.1 mg/L (final concentration 0.4 mM). All cell lines were grown under a humidified atmosphere of 5% CO2 at 37 °C.

### Patients and tissue specimens

Our study was performed using 233 paraffin-embedded cervical carcinoma samples, which were took from patients diagnosed at the Sun Yat-Sen University Cancer Center. These specimens were obtained from 140 patients with stage I, 90 patients with stage II, and two patients with stage III cervical cancer who received surgery and postoperative chemotherapy or radiation, according to pathological high-risk factors, from 2005 to 2010. None these patients received any anti-cancer therapy before surgery. Information on the clinical characteristics of the patients with cervical cancer is summarized in the Additional file 1: Table S1. For quantitative real-time reverse transcription polymerase chain reaction (qRT-PCR) and western blotting analysis, fresh cervical cancer and matched noncancerous cervical tissues samples were obtained from sevenpatients, and fresh cervical cancer samples were resected from six patients without metastasis and from eight patients with metastasis. Surgeries were performed at the Sun Yat-sen University Cancer Center between January 2017 and May 2017. Ethics approval and written informed consent for the use of the specimens were provided by the Institutional Research Ethics Committee.

### Gene set enrichment analysis (GSEA)

Gene Set Enrichment Analysis (GSEA) is a computational method that determines whether an a prior defined set of genes shows statistically significant, concordant differences between two biological states (e.g. phenotypes). The results presented in the current manuscript use the two phenotypes divided by the median of NUSAP1 mRNA expression of Cervical squamous cell carcinoma and endocervical adenocarcinoma (CESC) TCGA data. GSEA was performed using GSEA 2.0.9 (http://www.broadinstitute.org/gsea/) according to its guideline [28, 29]. The gene sets (the curated gene sets C2) were downloaded from GSEA 2.0.9 (http://www.broadinstitute.org/gsea/).

#### MTT assay

Cells were seeded in 96-well plates at initial density of (0.2 × 10^4^/well). At each time point, cells were stained with 100 μL sterile 3-(4, 5-dimethyl-2-thiazolyl)-2,5-diphenyl- 2H-tetrazolium bromide (MTT) dye (0.5 mg/mL, Sigma) for 4 h at 37 °C, followed by removal of the culture medium and addition of 150 μL of dimethyl sulphoxide (Sigma). The absorbance was measured at 490 nm using a microplate reader (Bio-Rad). The experiment was repeated three times.

### RNA extraction, reverse transcription, and quantitative real-time PCR

RNA extraction, reverse transcription, and real-time PCR were performed as described previously [25]. The primers were designed using primer Express Software v.2.0 and comprised: *NUSAP1* forward: 5′-CTGACCAAGACTCCAGCCAGAA-3′ and reverse: 5′-GAGTCTGCGTTGCCTCAGTTGT-3′; SRY-Box 2 (*SOX2*) forward: 5′-GCTACAGCATGATGCAGGACCA-3′ and reverse: 5′-TCTGCGAGCTGGTCATGGAGTT-3′; octamer-binding protein 4 (*OCT4*) forward: 5′-CCTGAAGCAGAAGAGGATCACC-3′ and reverse: 5′-AAAGCGGCAGATGGTCGTTTGG-3′; homeobox transcription factor Nanog (*NANOG*) forward: 5′-CTCCAACATCCTGAACCTCAGC-3′ and reverse: 5′-CGTCACACCATTGCTATTCTTCG-3′; B lymphoma Mo-MLV insertion region 1 homolog (*BMI1*) forward: 5′-GGTACTTCATTGATGCCACAACC-3′ and reverse: 5′-CTGGTCTTGTGAACTTGGACATC-3′; glyceraldehyde-3-phosphate dehydrogenase (*GAPDH*) forward: 5′-GTCTCCTCTGACTTCAACAGCG-3′ and reverse: 5′-ACCACCCTGTTGCTGTAGCCAA-3′; MYC forward: 5′-CGTCCTCGGATTCTCTGCTC-3′ and reverse: 5′-GCTGGTGCATTTTCGGTTGT-3′; matrix metalloproteinase 7 (*MMP7*) forward: 5′-TCGGAGGAGATGCTCACTTCGA-3′ and reverse: 5′-GGATCAGAGGAATGTCCCATACC-3′; matrix metalloproteinase 9 (*MMP9*) forward: 5′-GCCACTACTGTGCCTTTGAGTC-3′ and reverse: 5′-CCCTCAGAGAATCGCCAGTACT-3′; CD44 forward: 5′-CCAGAAGGAACAGTGGTTTGGC-3′ and reverse: 5′-ACTGTCCTCTGGGCTTGGTGTT-3′.All experiments were conducted three times. Additionally, *GAPDH* was chose as the internal control to normalize the expression levels of all the genes in the samples, and the fold changes were calculated using the relative quantification 2- [(cycle threshold (Ct) of gene)-(Ct of *GAPDH*)].

### Western blotting analysis

Western blotting assays [30] were conducted using anti-NUSAP1 rabbit polyclonal antibodies (SAB1407458, Sigma-Aldrich, St Louis, MO, USA), antibodies against p84(ab102684, Abcam), GAPDH (D16H11, Cell Signaling), α-tubulin (#3873, Cell Signaling), E-cadherin(#14472, Cell Signaling), vimentin(#5741, Cell Signaling), MYC (#2276, Cell Signaling), MMP7(#71031, Cell Signaling), MMP9(D603H, Cell Signaling), CD44(#37259, Cell Signaling), SUMO-1 (#4930, Cell Signaling),TCF4 (#2569, Cell Signaling), RanBP2 (ab2938, Abcam, Cambridge, MA) and β-catenin(#8480,1:1000, Cell Signaling). GAPDH, α-tubulin, or P84 were used as the endogenous controls.

### Immunoprecipitation

Lysates were prepared from Siha cells using lysis buffer (150 mM NaCl, 10 mM HEPES, pH 7.4, 1% NP-40). Lysates were then incubated with HA affinity agarose (Sigma-Aldrich), or NUSAP1 or RanBP2 antibody with protein G agarose, overnight at 4 °C. Beads containing affinity-bound proteins were washed 6 times by immunoprecipitation wash buffer (150 mM NaCl, 10 mM HEPES, pH 7.4, 0.1% NP-40), followed by elutions 1 M glycine (pH 3.0). The eluates were then mixed with sample buffer and denatured, and used for the western blot analysis.

### Immunohistochemistry (IHC)

Immunohistochemical staining was carried on the 233 paraffin-embedded cervical carcinoma tissues using anti-NUSAP1 antibodies (HPA024904,1:600, Sigma) and antibodies against β-catenin(#8480,1:1000, Cell Signaling). The degree of immunostaining was reviewed and assessed in a blinded manner by two independent pathologists. We defined the score of cell proportions as follows: 0, no positive cells; 1, < 10% positive cells; 2, 10–35% positive cells; 3, 35–75% positive cells; 4, > 75% positive cells. Staining intensity was graded using the following standard: 0, no staining; 1, weak staining (light yellow); 2, moderate staining (yellow brown); 3, strong staining (brown). The staining intensity (SI) was evaluated multiplying the staining intensity score by the proportion of positive tumor cells. During the assessment, we used the SI to evaluate the NUSAP1 and nuclear β-catenin protein expression levels. The possible scores were 0, 1, 2, 3, 4, 6, 8, 9, and 12. Specimens with a score ≥ 6 were defined as having high NUSAP1 or nuclear β-cateninexpression, and samples with a score < 6 were defined as having low NUSAP1 or nuclear β-catenin expression.

### Plasmids, retroviral infection, and transfection

The NUSAP1 cDNA was amplified using PCR and subcloned into vector pMSCV-puro-NUSAP1. We cloned two short hairpin RNA (shRNA) oligonucleotides to produce pSuper-puro-NUSAP1-shRNA to knockdown endogenous *NUSAP1*(RNA#1: 5′-GCACCAAGAAGCTGAGAATGC-3′,and RNA#2:5′- GGAAATGGAGTCCATTGATCA-3′). The Lipofectamine 3000 reagent (Invitrogen) was used to transfect the plasmids into cells, following the manufacturer’s instructions. Stable cell lines expressing *NUSAP1* or *NUSAP1* shRNA were selected for 10 days by treatment with 0.5 μg/ml of puromycin for 48 h after infection. The sequence of RanBP2 siRNA was GAAUAACUAUCACAGAAUG .

### Wound healing assay

Six-well plates were seeded with cells transfected with vector, *NUSAP1*, shRNA-vector, or *NUSAP1* shRNA and incubated under suitable conditions until 90% confluence was reached. Wounds were induced by scratching the confluent cells using a pipette tip after 48 h of serum starvation. The cells were washed with phosphate-buffered saline (PBS) three times and then incubated in RPMI-1640 medium. At the indicated times (including time 0), the wounds were photographed under an inverted Olympus IX50 microscopeand measured. Each experiment was performed at least three times.

### Invasion assay

The invasion assay was conducted using aTranswell chamber with an 8-mm membrane filter insert (Corning) with Matrigel (BD,Biosciences). Briefly, the indicated cells were cultured in serum-free medium. The cells were placed into the upper chamber, and the lower chamber was supplied with 1 ml of medium containing 10% FBS. After 48 h of incubation at 37 °C, the cells in the upper chamber were gently removed using a cotton swab. The migratedcells on the lower membrane surface were fixed in 1% paraformaldehyde, stained with hematoxylin, and counted (ten random fields per well; 100× magnification). The count number was represented as the mean number of cells per field of view. All the experiments were conducted in triplicate andthe data are presented as the mean ± standard deviation (SD).

### Sphere formation assays

The indicated cells were implanted into six-well ultra-low attachment plates. Cells were incubated in the Dulbecco’s modified Eagle’s medium (DMEM)/F12 serum-free medium (Invitrogen) with 20 ng/ml epidermal growth factor (EGF), 2% B27 (Invitrogen), 5 μg/ml insulin (Sigma-Aldrich), 0.4% bovine serum albumin (Sigma-Aldrich), and 20 ng/ml basic fibroblast growth factor (bFGF; PeproTech). After 10 days of incubation, the number of spheres was calculated and their volume was assessed on a BX-X700 fluorescence microscope (Keyence, Osaka, Japan). The experiment was carried out three times.

### Side population analysis

To analyze the side population cells proportion, the cell suspensions were labeled with Hoechst 33,342 (Sigma-Aldrich) dye for side population analysis as per standard protocol [31, 32]. Briefly, cells were resuspended at EMEM medium (ATCC-30-2003) containing 2%FBS (Gibco, USA) at a density of 10^6^/mL. Hoechst 33,342 dye was added at a final concentration of 5 Ig/ml in the presence or absence of verapamil (Sigma-Aldrich) and the cells were incubated at 37 °C for 90 min with intermittent shaking. At the end of the incubation, the cells were washed with EMEM medium adding 2%FBS and centrifuged down at 4 °C, and resuspended in ice-cold EMEM medium. Propidium iodide (Sigma, USA) at a final concentration of 2 Ig/mL was added to cells to gate viable cells. The cells were filtered through a 40-lm cell strainer to obtain single cell suspension before sorting. Analysis and sorting was done on a FACS AriaI (Becton Dickinson). The Hoechst 33,342 dye was excited at 355 nm and its dual-wavelength emission at blue and red region was plotted to get the SP scatter.

### Immunofluorescence imaging

The indicated cells were placed on 24-well plate and then incubated at 37 °C in 5% CO_2_ overnight. The cells were fixed with 4% paraformaldehyde and permeabilized with 0.1% Triton X-100 before being blocked with 1% BSA in PBS buffer for 1 h the next day. Cells were incubated with primary antibodies against E-cadherin (1:400) (24E10, Cell Signaling) and vimentin (1:50) (D21H3,Cell Signaling),whichwere conjugated with Alexa Fluor 488 and Alexa Fluor 555, respectively, at room temperature for 1 h in the dark. Cell nuclei were stained with 4,6 diamidino-2-phenylindole (DAPI, Sigma) for 3 min. The cells were then visualized and photographed under a confocal laser-scanning microscope (Olympus FV1000, Japan).

### Luciferase assay

Cells were implanted in 48-well plates and incubated for 24 h. One nanogram of the pRL-TK Renilla plasmid (Promega) containing the TOP-Flash or FOP-Flash luciferase reporter were transfected into the cells using the Lipofectamine 2000 Reagent (Invitrogen). A Dual-Luciferase Reporter Assay kit (Promega) was used to detect the luciferase activity of the indicated cells according to the manufacturer’s instructions.

### Animal experiments

BALB/c-nu mice (female, 3–5 weeks old; weighing 11–13 g) were purchased from Hunan SJA Laboratory Animal Co. Ltd. (Changsha, China). All experimental procedures were approved by the Institutional Animal Care and Use Committee of Sun Yat-sen University. As for thelung metastasis colonization mousemodels, the BALB/c-nu mice were randomly separated into four groups (*n* = 6/group) and injected with 5 × 10^5^cells (SiHa-vector/SiHa-NUSAP1;SiHa-shRNA-Vector/SiHa-NUSAP1-shRNA#1) through their tail veins. The Xenogen IVIS spectrum imagining system (Caliper) was used to assess the bioluminescence of tumor colonization and growth in the lung and popliteal lymph node tissues. At the end of the experiment (45 days), the mice were euthanized and their lungs were excised, fixed in formalin, and paraffin-embedded for the hematoxylin and eosin staining. The numbers of lung surface metastatic nodules were calculated under a dissecting microscope and expressed as the mean ± standard error of the mean (SEM). As for the lymph node metastatic mouse model, the BALB/c-nu mice were randomly divided into four groups (*n* = 6/group). The NUSAP1, NUSAP1-RNA#1 or vector-transduced SiHa cells (1.5 × 10^5^), which stably express firefly luciferase, were inoculated into the foot-pads of the mice. The mice were sacrificed on day 45, and the primary tumours and popliteal lymph nodes were excisedand paraffin embedded. Serial 4.0 mm sections were taken and analysed by IHC with anti-luciferase antibodies (Abcam). All animal studies were conducted withthe approval of the Medical Experimental Animal CareCommission of Sun Yat-sen University Cancer Center.

### Bioinformatics and data analysis

NUSAP1 mRNA expression in normal cervix and cervical cancer tissues were examined based on The Cancer Genome Atlas (TCGA; https://gdc.cancer.gov/) and the Gene Expression Omnibus (GEO) dataset (GSE7803 and GSE9750) (see URL https://www.ncbi.nlm.nih.gov/geo). Wedownloaded the SeriesMatrix File. Gene expression was presented as the mean value of multiple probes for eachgene after log2 transformation. Comparisons (normal cervix tissues versus cervical cancertissues) were analyzed by Mann-Whitney U test. *P* < 0.05 was considered statistically significant.

### Statistical analysis

The statistical analyses were conducted using the SPSS 16.0 statistical software package (IBM, Armonk, NY, USA). Kaplan–Meier analysis was applied to establish survival curves and the log-rank test was used to evaluate the statistically significant differences. Student’s t-test (two-tailed) was performed to compare the continuous data. In addition, other statistical methods for data analysis included Fisher’s exact test, chi-squared (χ^2^) test, Mann-Whitney U test, Spearman correlation analysis, and multivariate cox regression analysis. *P* value of < 0.05 was regarded as statistically significant in all cases.

## Results

### Expression of NUSAP1 in cervical cancer

**Fig. 1:**
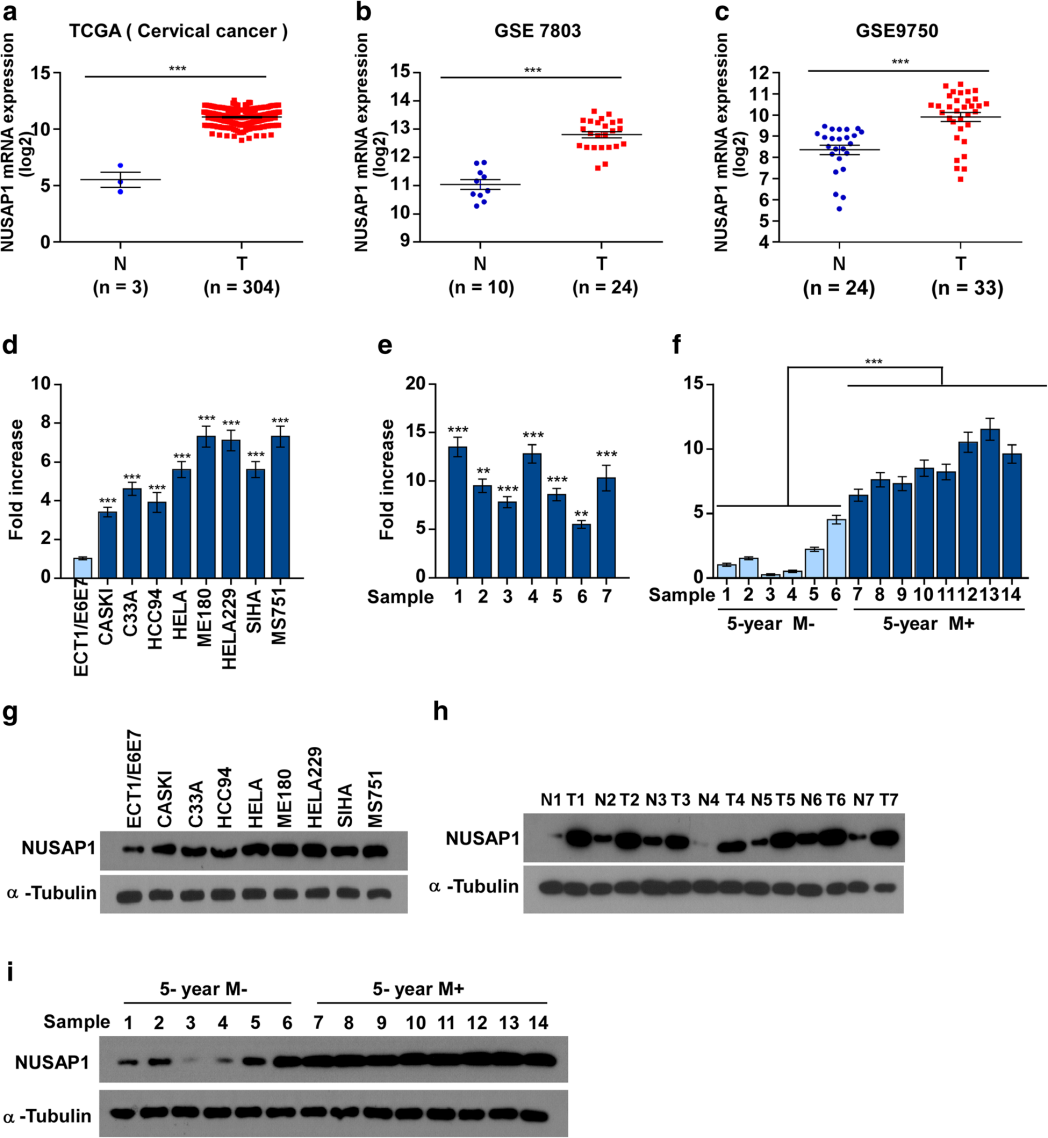
NUSAP1 is upregulated in cervical cancer. **a** NUSAP1 mRNA expression levels were significantly upregulated in cervical cancer tissues compared with normal tissues, as indicated by The Cancer Genome Atlas (COAD) cervical cancer dataset and public GEO cervical cancer datasets GSE7803 and GSE9750. **d**, **e** Real-time PCR analysis of NUSAP1 mRNA in ECT1/E6E7 human immortalized cervical epithelial cells and in eight cultured cervical cancer cell lines (**d**) and seven fresh primary cervical cancer tissues as compared with paired normal tissues (**e**). **f** Real-time PCR analysis of NUSAP1 mRNA in six cervical cancer samples without metastasis and eight cervical cancer samples with metastasis within 5 years; GAPDH was used as a loading control. **g**, **h** Western blotting analysis of NUSAP1 expression in ECT1/E6E7 human immortalized cervical epithelial cells and eight cultured cervical cancer cell lines (**g**), and in seven paired fresh primary cervical cancer tissues and adjacent non-cancerous tissue sections (**h**). **i** Western blotting results of NUSAP1 expression in six cervical cancer cases without metastasis and eight cervical cancer specimens with metastasis within 5 years; α-tubulin was used as a loading control. All experiments were performed in triplicate. Results are the mean ± SD. **p < 0.01; ***p < 0.001

Microarray analysis (The Cancer Genome Atlas (TCGA) (Cervical squamous cell carcinoma and endocervical adenocarcinoma (CESC) TCGA data), GSE7803 and GSE9750) revealed that *NUSAP1* was upregulated in cervical cancer samples compared with that in normal cervix tissues (Fig. 1a-c). To explore the role of NUSAP1 in cervical cancer, we selected seven paired cervical carcinoma tissues and eight cervical cancer cell lines to detect its expression. Western blotting assay and qRT-PCR assays showed that NUSAP1 was upregulated in the seven fresh primary cervical cancer specimens compared with that in the paired normal samples, and NUSAP1 was over expressed in the eight cervical cancer cell lines compared with that in the human cervical immortalized squamous cell line (Ect1/E6E7) (Fig. 1d,e,g,h). Furthermore, the expression of NUSAP1 was evaluatedin those samples that with 5-year metastasis compared with those samples that without metastasis within 5 years (Fig. 1f, i).

### Clinical significance of NUSAP1 in cervical cancer

**Fig. 2:**
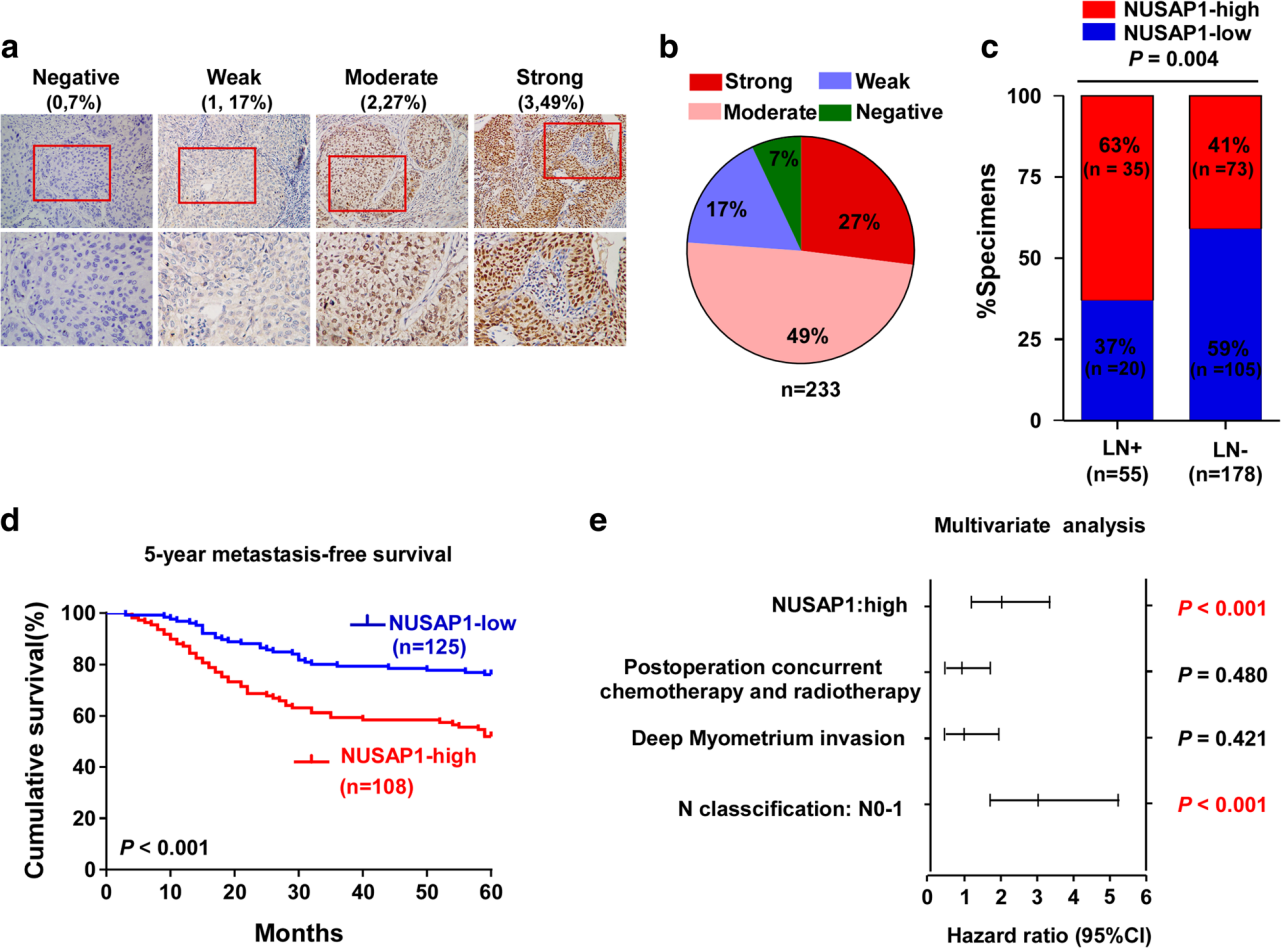
Upregulation of NUSAP1 is associated with poor prognosis in cervical cancer. **a** Representative images of NUSAP1 staining in cervical cancer, which was scored as strong + 3, moderate + 2, weak + 1, and negative 0 (SP, × 200 and × 400). The proportion of each staining score is indicated (brackets). Bottom row shows the magnified inset area. **b** The number of cases with all staining intensities was indicated. **c** Correlation between 5-year metastatic relapse and NUSAP1 expression in patients. Chi-square test was used. **d** Kaplan–Meier metastasis free survival curves for cervical cancer patients stratified by high (*n* = 113) versus low (*n* = 120) NUSAP1 expression. *P*-values were calculated using the log-rank test; *P* < 0.001. **e** Multivariate analysis of clinical variables in cervical cancer using a Cox-regression model

The clinical significance of NUSAP1 was further assessed by immunohistochemistry (IHC) staining in 233 archived cervical cancer samples (Additional file 1: Table S1 and Additional file 2: Table S2). The staining intensities of NUSAP1 expression divided into four degrees: negative (0 score, 7%), weak (1 score, 17%), moderate (2 score, 49%), and strong (3 score, 27%) (Fig. 2a). The number of cases with all staining intensities was indicated in Fig. 2b. Correlation analysis showed that high expression of NUSAP1 positively and significantly correlated with lymph node metastasis in patients (Fig. 2c). Importantly, Kaplan–Meier survival curves and log-rank tests revealed that patients with high NUSAP1 expression had shorter 5-year metastasis-free survival (*P* < 0.001, Fig. 2d). Additionally, multivariate Cox regression analysis found that the NUSAP1 protein expression level and N classification were independent prognostic indicators for cervical cancer (Fig. 2e).

### Influence of expression levels of NUSAP1 on migration and invasion capacity of cervical cancer cells both in vitro and in vivo

**Fig. 3:**
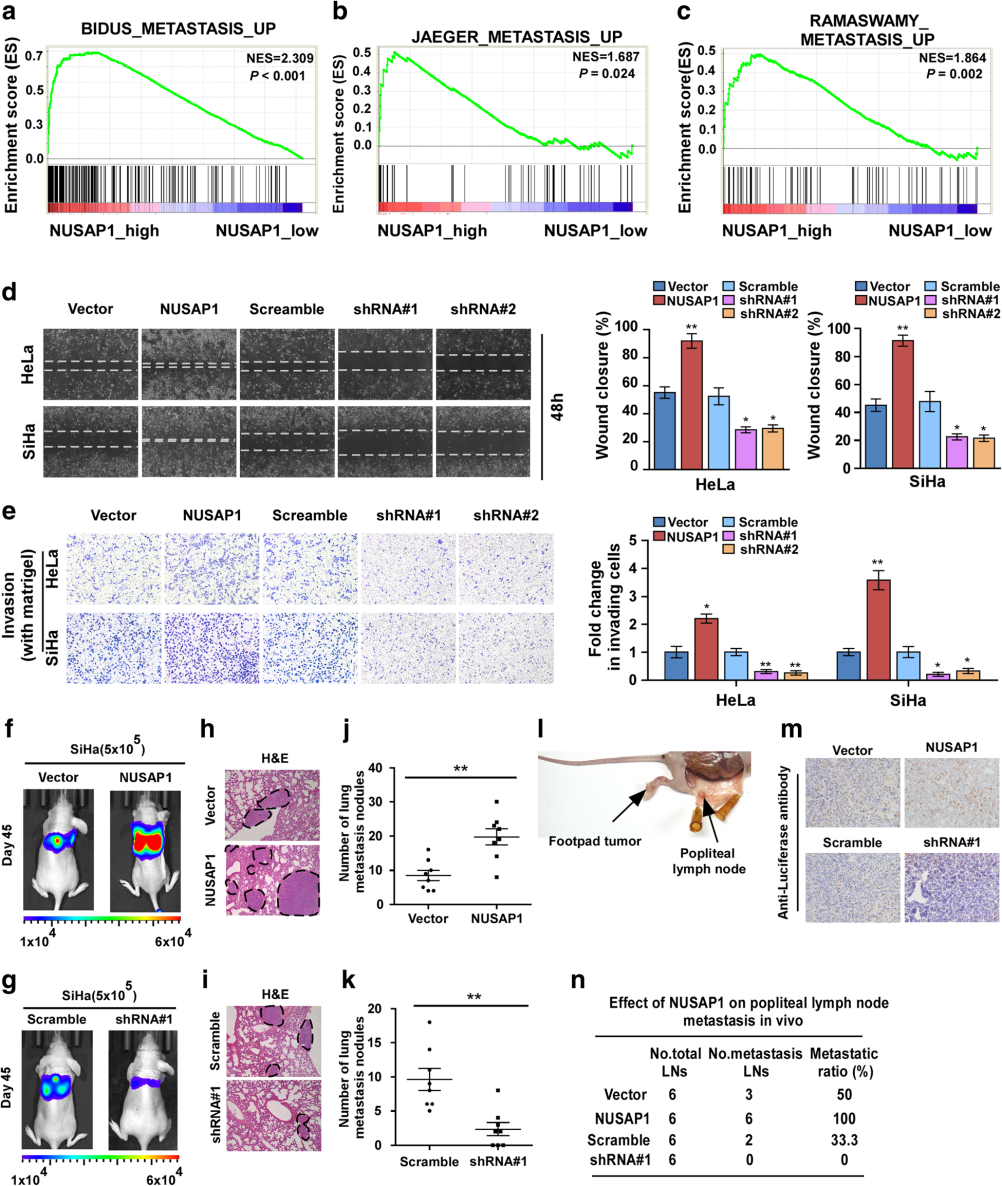
NUSAP1 overexpression promotes metastasis and invasion in cervical cancer cells. **a**-**c** GSEA plot showing that NUSAP1 expression positively correlated with metastasis. **d** Representative images (left panel) and quantification (right panel) of wound-healing assays for the indicated cell lines. Wound closure was photographed at 0 and 48 h after wounding. **e** Representative micrographs and quantification of the invasiveness of indicated cells in matrigel-coated transwell assays. **f**-**k** In vivo metastasis assays of indicated Siha cells, the lung metastasis burden of xenografted animals was monitored weekly using bioluminescent imaging (BLI). Shown are BLI images of representative mice on day 45 after injection. The color scale depicts the photon flux (photons per second) emitted from the metastatic cells (in left panel). Representative H&E stained lung sections and the number of lung metastatic nodules in the tested mice (*n* = 6). (in middle panel). The number of metastatic nodules formed in the lungs is summarized in the right panel. **l** A popliteal lymph node metastasis model was established by footpad inoculation in mice and analyzed. **m** Representative micrographs of the popliteal lymph nodes immunostained with anti-luciferase antibody. **n** Ratios of metastatic to total dissected popliteal lymph nodes from mice inoculated with the indicated cells. All experiments were performed in triplicate. Results are the mean ± SD. *p < 0.05; **p < 0.01

We analyzed the biological role of NUSAP1 in metastasis of cervical carcinoma by gene set enrichment analysis (GSEA) on the basis of mRNA expression data from TCGA of CESC samples. The GSEA results showed that overexpression of *NUSAP1* was positively associated with metastasis (Fig. 3a-c) (Additional file 3: Figure S4(A-G) and Additional file 4: Figure S5(A-E)). To further explore the function of NUSAP1 in cervical cancer metastasis, we created stable *NUSAP1*-overexpressing and -knockdown cell lines using HeLa and SiHa cervical cancer cells (Additional file 5: Figure S1A). As shown in Fig. 3d-e, wound healing assays and the Matrigel-coated transwell assays showed that NUSAP1 upregulation markedly increased the cell migration and invasion ability, while knockdown of *NUSAP1*caused an apparent decrease in cell migration and invasion. We then evaluated the effect of NUSAP1 on cervical carcinoma metastasis in vivo by lung colonization models and popliteal lymph node metastasis model. SiHa-NUSAP1 and SiHa-NUSAP1-shRNA#1cells were injected into the tail vein or footpads, while SiHa-Vector and SiHa-shRNA-Vector cells were used as controls (*n* = 6/group). Mice were sacrificed on day 45, and the lung and popliteal lymph nodes were enucleated and analyzed. Strikingly, our results revealed that NUSAP1 strongly enhanced the metastasis of SiHa cells, which was further confirmed by counting the visible metastatic lesions and H&E staining (Fig. 3 h-k). Meanwhile, we found that the lymph nodes in tumours formed from NUSAP1-transducted cells displayed higher numbers of luciferase-positive tumour cells than tumours formed vector-control cells. Conversely, the lymph node formed from NUSAP1-silenced cells had less luciferase-positive tumour cells than vector-control tumours (Fig. 3l,m). Strikingly, the ratio of metastatic to total dissected popliteal lymph nodes were obviously higher in the SiHa-NUSAP1 group (100% (6/6)) than in the vector groups (50% (3/6)). By contrast, there were no metastatic lymph nodes were found in the NUSAP1-silenced groups (Fig. 3n). To investigate whether NUSAP1 affect the proliferation rates of cervical cancer Hela and Siha cell lines. MTT assay shows that overexpression of NUSAP1 or knockdown of NUSAP1 did not significantly affect proliferation rates of cervical cancer Hela and Siha cell lines (Additional file 6: Figure S2A-D). Consistent with the cells proliferation results in vitro, the indicated cells (Siha/Vector, Siha/NUSAP1,Siha/Scramble and Siha/shRNA#1 cells) were also subcutaneously inoculated into the node mouse (*n* = 6/group). As shown in the Additional file 6: Figure S2E-G the subcutaneous tumors size formed from NUSAP1-ovexpression group and NUSAP1-silenced group has no significant difference. Collectively, these results reveal that NUSAP1 promotes cervical cancer cells metastasis both in vitro and in vivo.

### NUSAP1 promotes epithelial-mesenchymal transition

**Fig. 4:**
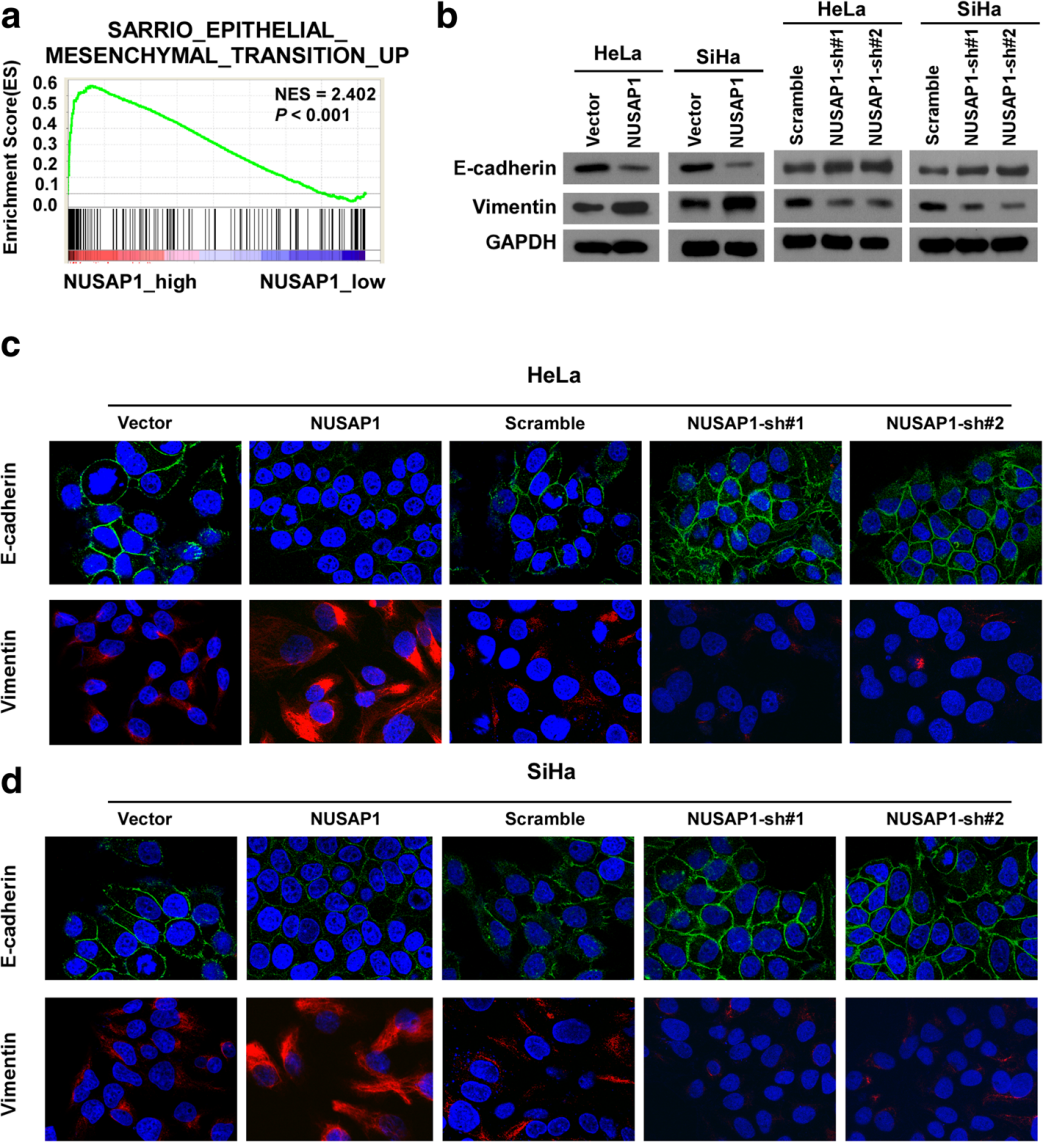
Upregulation of NUSAP1 promotes an EMT-like phenotype in cervical cancer cell lines. **a** GSEA plot showing that NUSAP1 expression positively correlates with EMTin published cervical cancer gene expression profiles (TCGA). **b** EMT markers, such as E-cadherin and vimentin, were analyzed by western blotting in the indicated cells. GADPH was used as the loading control. **c**, **d** Immunofluorescence staining for E-cadherin and vimentin in the indicated cells (× 200)

Previous studies indicated that EMT is a critical step for tumor relapse and metastasis [16, 33]. The GSEA plot showed that NUSAP1 expression correlated positively with EMT (*P* < 0.001; Fig. 4a). Western blotting and immunofluorescent staining demonstrated that high expression of *NUSAP1*markedly reduced the expression of the epithelial marker E-cadherin and increased the expression level of the mesenchymal marker vimentin compared with that in the control group (Fig. 4b-d). Conversely, E-cadherin expression was upregulated and vimentin expression was downregulated in *NUSAP1*-silenced cells compared with the vector cells. Taken together, these results showed that NUSAP1 promotes EMT in cervical cancer cells.

### NUSAP1 promotes the CSCs properties in cervical cancer

**Fig. 5:**
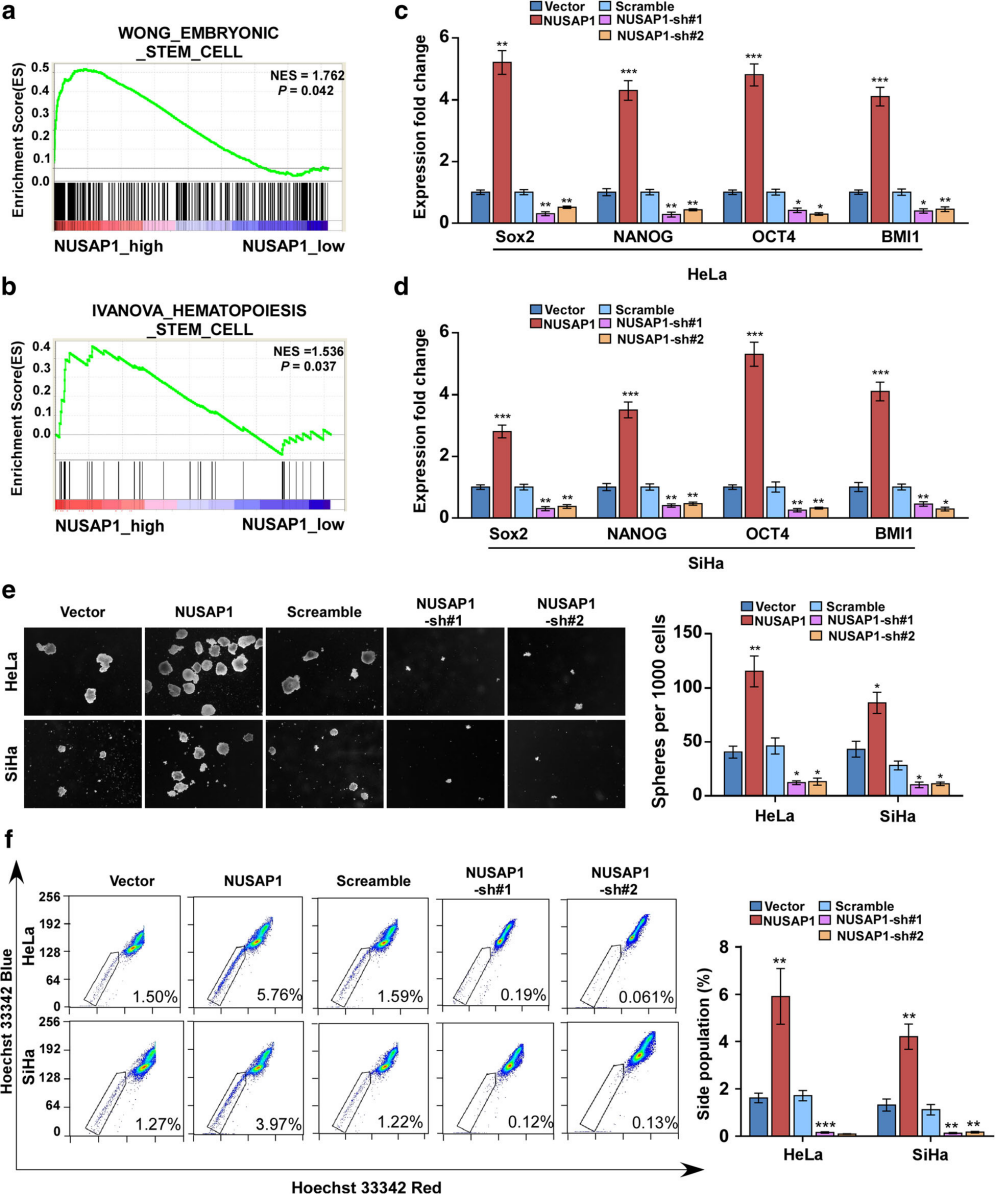
NUSAP1 enhances CSCs properties. **a**, **b** GSEA of TCGA database showed significant enrichment of stemness gene modules (WONG_EMBRYONIC_STEM_CELL,IVANOVA_HEMOTOPOIESIS_STEM_CELL), in samples with high expression of NUSAP1. NES, normalized enrichment score. **c**, **d** Real-time PCR analysis of mRNA expression of multiple pluripotency-associated factors, including Sox2, NONOG, OCT4 and BMI1 in the indicated cells. **e** Representative images of spheres formed by the indicated cells. Scale bar = 100 μm. Histograms showing the mean numbers of spheres are on the right-hand side. **f** Hoechst 33342 dye exclusion assay showing that overexpression of NUSAP1 increased, whereas silencing of NUSAP1 decreased, the proportion of SP cells among the indicated cells. Histograms showing the mean percentages of side population cells are on the right-hand side. All experiments were performed in triplicate. Results are the mean ± SD. *p < 0.05; **p < 0.01; ***p < 0.001

Previous studies showed that CSCs have critical roles in the progression and metastasis of multiple types of cancer [22]. To assess whether NUSAP1 promotes the CSC properties of cervical cancer cells, we first analyzed the GSEA based on mRNA expression data from TCGA of CESC samples.. The results showed significant enrichment stem cell gene modules, in samples with high NUSAP1 (*P* = 0.042; *P* = 0.037; Fig. 5a, b). Furthermore, we explored the transcription levels of multiple pluripotency-associated markers, including *SOX2*, *NANOG*, *OCT4*, and *BMI1* both in HeLa and SiHa cells. In both cell lines, the expression levels of these genes were increased in *NUSAP1* overexpressing cells and downregulated in NUSAP1-silenced cells (Fig. 5c, d). Moreover, we also performed tumor sphere formation assays to detect the effect of NUSAP1 on the self-renewal of cervical cancer cells. As expected, we found that *NUSAP1* upregulation promoted tumor sphere formation of SiHa and HeLa cells, generating nearly 3-fold more spheres compared with control cells. The tumor sphere formation obtained from NUSAP1-silenced cells generated approximately 3-fold fewer spheres compared with control cells (Fig. 5e). Moreover, flow cytometry assays showed that a smaller proportion of side population (SP) cells in the NUSAP1-silenced SiHa and HeLa cells compared to the control cells, while the proportion of SP cells was significantly increased in NUSAP1-upregulated cells compared to controls (Fig. 5f). These results suggest that NUSAP1 is essential for the maintenance of cervical cancer CSCs properties.

### NUSAP1 activates Wnt/β-catenin pathway in cervical cancer cells

**Fig. 6:**
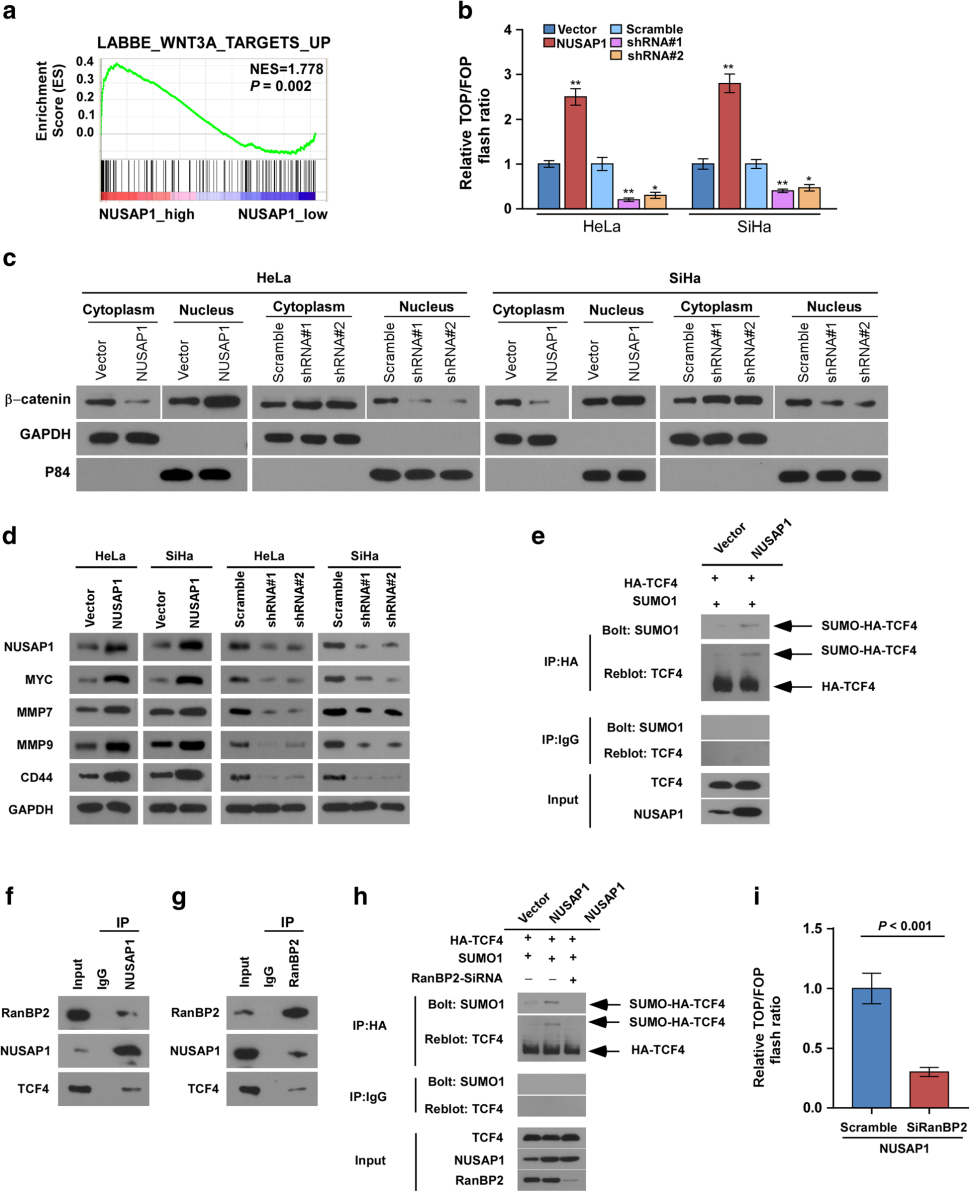
NUSAP1 contributes to the activation of canonical Wnt/β-catenin signaling. **a** GSEA plot showing that NUSAP1 expression positively correlates with Wnt/β-catenin-activated gene signatures (LABBE_WNT3A_TARGETS_UP). **b** TOP/FOP luciferase assay of TCF/LEF transcriptional activity in the indicated cells. **c** Western blotting analysis of β-catenin expression in the cytoplasm and nucleus of the indicated cells. GADPH and p84 were used as loading controls for the cytoplasmic and nuclear fractions, respectively. **d** Western blotting analysis of the protein expression levels of the β-catenin target genes in the indicated cells. GADPH was used as the loading control. **e** Lysates from Siha cells transfected with HA-TCF4 and Flag-SUMO1 were immunoprecipitated with anti-HA affinity agarose, followed by western blot analysis of TCF4 and SUMO1. **f**, **g** Lysates of Siha cells were immunoprecipitated using NUSAP1 or RanBP2 antibodies, and the interactions of NUSAP1 with TCF4 and RanBP2 were analysed by immunoblotting. **h** Siha cells were transfected with the indicated control RNA and siRNA against RanBP2. Lysates were immunoprecipitated with anti-HA antibody, followed by western blot analysis of TCF4 and SUMO1. **i**. TOP/FOPluciferase assay of TCF/LEF transcriptional activity in the NUSAP1-ovexxpressed Siha cells transfected with the indicated control RNA and SiRNA against RanBP2.All experiments were performed in triplicate. Results are the mean ± SD. *p < 0.05; **p < 0.01; ***p < 0.001

To further explore the mechanism by which NUSAP1 promotes metastasis of cervical cancer cells, we analyzed publicly available gene expression array data for cervical cancer using GSEA. We found that *NUSAP1* overexpression was positively related to Wnt/β-catenin signaling (*P* = 0.002; Fig. 6a). To explore the effect of NUSAP1 on the Wnt/β-catenin pathway, we transfected TOPflash and FOPflash constructs and the RenillaPrl-TK plasmid as an internal controlinto Hela and Siha cells. We found that NUSAP1 elevation remarkably increased β-catenin/TCF transcriptional activity (Fig. 6b). By contrast, knockdown of NUSAP1 significantly repressed the β-catenin/TCF transcriptional activity. Next, we explored the effect of NUSAP1 on the subcellular localization of β-catenin. Consistent with our hypothesis, western blotting analysis of nuclear and cytoplasmic cellular fractions showed that overexpression of NUSAP1 induced, whereas knockdown of NUSAP1 impaired, the translocation of β-catenin into the nucleus (Fig. 6c). Meanwhile, we observed that upregulation of NUSAP1 in Hela and Siha cells markedly enhanced the transcription and translation of representative β-catenin target genes, including *MYC*, *MMP7*, *MMP9*, and *CD44*. In contrast, silencing of *NUSAP1* reduced the expression levels of the representative β-catenin target genes (Fig. 6d, Additional file 7: Figure S3A-B). We next examined whether TCF4 protein is SUMOylated by RanBP2. Siha cells were transfected with TCF4 and SUMO1 cDNA and analyzed by immunoprecipitation and immunobloting. As shown in the Fig. 6e, NUSAP1 overexpression enhanced the SUMOylation of TCF4 compared to the vector group. Further work is needed to elucidate the details of mechanism whereby SUMOylation promotes the functional activity of Wnt/β-catenin signaling. We tested whether SUMOylation E3 ligase (RanBP2) co-IPed with endogenous NUSAP1 in cervical cancer cells. The identity of these proteins was detected by immunoblotting using available antibodies. RanBP2 and TCF4 were present in the immunoprecipitate with the anti-NUSAP1 antibody but not with control IgG (Fig. 6f). Moreover, NUSAP1 and TCF4 protein was detected in the immunoprecipitate with the anti- RanBP2 (Fig. 6g). Furthermore, knockdown of RanBP2 eliminated the SUMO conjugated form of TCF4 in NUSAP1-overexpressed Siha cells (Fig. 6h). Consistent with the previous results, We found that knockdown of RanBP2 remarkably repressed β-catenin/TCF transcriptional activity (Fig. 6i).

### The Wnt/β-catenin signaling pathway is required for NUSAP1-mediated cervical cancer cells metastasis and clinical relevance of NUSAP1 and β-catenin activation in human cervical carcinoma

**Fig. 7:**
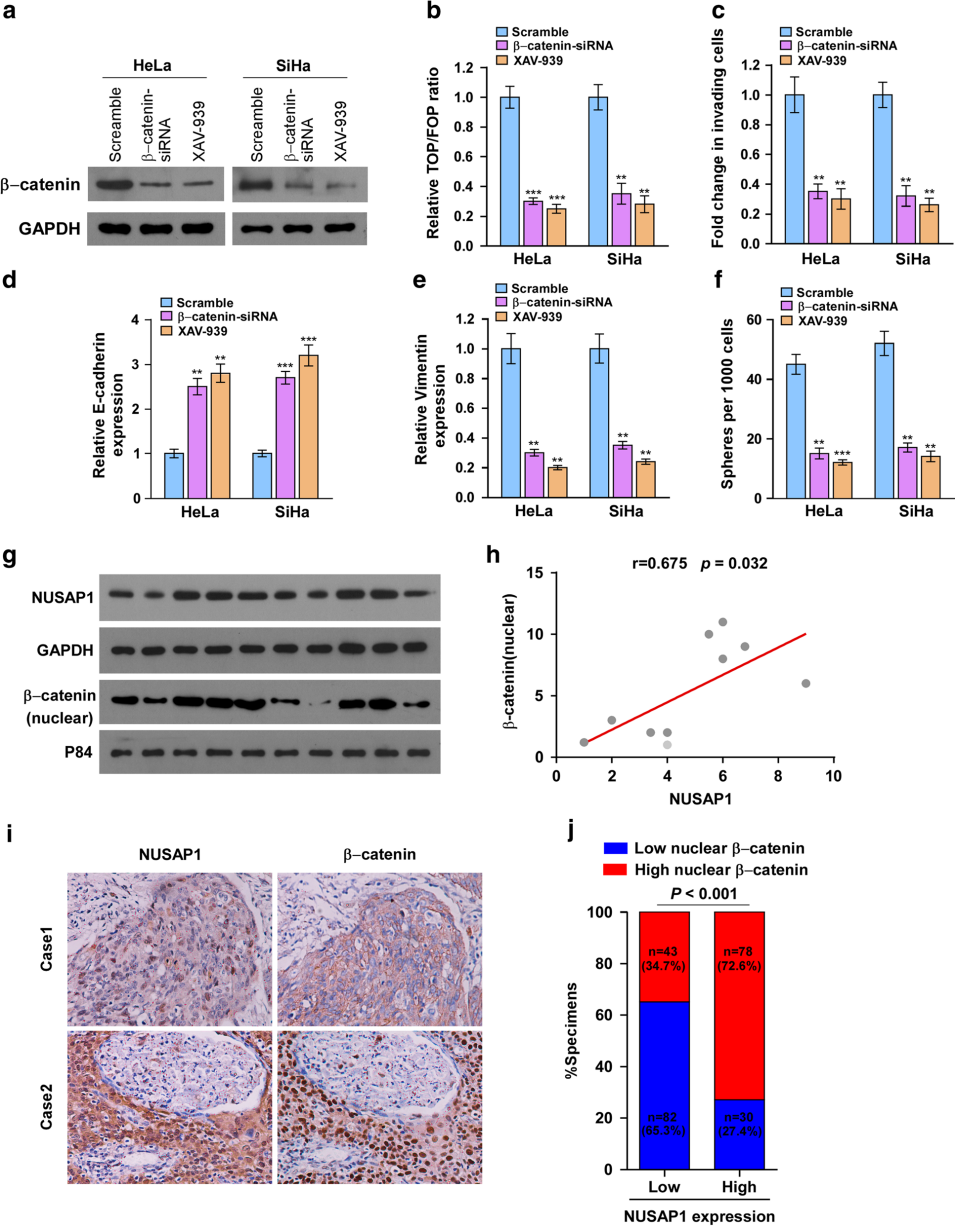
Canonical Wnt/β-catenin signaling mediates the effects of NUSAP1 and clinical relevance of NUSAP1-induced β-catenin activation in cervical cancer. **a** Western blotting analysis confirming the depletion of β-catenin in NUSAP1-transduced cells using a β-catenin-siRNA or XAV-939. **b** TOP/FOP luciferase activity in the indicated cells (**c**) Quantification of the invading cells in a matrigel-coated transwell assay. **d**, **e** Real-time PCR analysis of EMT markers such as E-cadherin (**d**) and vimentin (**e**) in the indicated cells. **f** Histograms showing the mean number of spheres formed by the indicated cells. **g**, **h**. Western blotting analysis of NUSAP1 and nuclear β-catenin protein levels in ten fresh cervical cancer tissues. GADPH and p84 were used as loading controls for the cytoplasmic and nuclear fractions, respectively. Right panel, correlation analyses between the protein expression of NUSAP and nuclear β-catenin in ten fresh cervical cancer samples. **i**, **j**. Immunohistochemical analysis of NUSAP1 and nuclear β-catenin protein levels in cervical cancer tissues. Left panel, images of two representative cases. Right panel, percentages of cervical cancer specimens with low or high expression of NUSAP1 relative to nuclear β-catenin expression levels. Results are the mean ± SD. **p < 0.01; ***p < 0.001

To further verify whether β-catenin activation was responsible for the NUSAP1-mediated effects, we detected the impact of blocking Wnt/β-catenin pathway on the metastasis and self-renewal capability of cervical cancer cells using the tankyrase inhibitor XAV-939(which was proved to stabilizes axin, a component of the β-catenin destruction complex, and widely used forblocking Wnt pathway) and a β-catenin-short interfering siRNA (Fig. 7a). As shown in Fig. 7b,both XAV939 and β-catenin siRNA treatments reduced TOP/FOP luciferase activities. Moreover, NUSAP1-induced invasion, EMT, and stemness in cervical cancer cells were repressed by the XAV939 or β-catenin siRNA treatments (Fig. 7c-f). To assess the clinical relevance of NUSAP1 and β-catenin, western blotting analysis was performed to evaluate the protein levels of NUSAP1 and β-catenin in ten fresh cervical carcinoma tissues. As shown in Fig. 7g and h, NUSAP1protein expression was positively associated with nuclear β-catenin protein expression (*P* = 0.032, *r* = 0.675). Moreover, immunohistochemical staining showed that NUSAP1 protein expression was positively and closely associated with nuclear β-catenin protein expression in the 233 paraffin-embedded cervical cancer tissues(*P* < 0.001; Fig. 7i, j). Collectively, these data suggested that the Wnt/β-catenin pathway is required for NUSAP1-mediated metastasis in cervical cancer cells and *NUSAP1* enhanced nuclear β-catenin protein expression and then evoked Wnt/β-catenin signaling which resulted in promotion of metastasis in cervical cancer samples.

## Discussion

In the present study, we showed that NUSAP1 was overexpressed in cervical cancer and correlated with unfavorable clinical outcome, especially via its critical role in promoting metastasis. NUSAP1 was associated with enhanced metastatic potential, both in vitro and in vivo. Moreover, increased levels of NUSAP1 were associated with CSCs properties of cervical cancer. In addition, our results indicated that NUSAP1 promotes metastasis of cervical cancer cells by inducing the sumoylation of TCF4 via interact with SUMO-E3 ligase Ran-binding protein2 (RanBP2) and then enhance the Wnt/β-catenin signaling activity. Taken together, the results presented a novel mechanism by which NUSAP1 persistently activates Wnt/β-catenin signaling, demonstrating the significant role of NUSAP1 in the metastasis of cervical cancer cells.

Recently, the important roles of NUSAP1 in tumorigenesis have been revealed in several types of tumor. NUSAP1 expression was correlated with *BRCA1*, *BRCA2*, and *BARD1* expression, and upregulation of NUSAP1 predicted poor prognosis in triple-negative breast cancer [11, 34]. Okamoto [35] demonstrated that inhibition of NUSAP1 suppressed tumor proliferation and enhanced the anti-tumor effect of paclitaxel by activating apoptotic pathways in oral squamous cell carcinoma. NUSAP1 has been reported as a potential prognostic marker for patients with glioma, and knockdown of *NUSAP1* suppressed the proliferation of glioma cells by inducing G2/M phase cell cycle arrest and apoptosis [36]. In colorectal cancer, NUSAP1 is an independent prognostic biomarker and its depletion induced cell apoptosis, and inhibited cell migration, cell invasion, cell proliferation, and EMT by inhibiting the expression of DNA methyltransferase 1 (DNMT1) [8]. Moreover, NUSAP1 expression has a significant role in the prediction of clinical outcome in hepatocellular carcinoma, in which NUSAP1regulates cellular proliferation, migration, and growth [37]. These studies indicated that NUSAP1 might have an important role in enhancing the malignant properties of human cancers, which leads to poor patient prognosis. In the present study, we revealed the oncogenic role and prognostic significance of NUSAP1 in cervical cancer, which agreed with the findings detailed above. Furthermore, we showed that overexpression of *NUSAP1*was markedly associated with lymph node metastasis and promoted cervical cancer cells metastasis by activating the Wnt/β-catenin pathway. Thus, our study provides evidence for the clinical and biological significance of NUSAP1 in cervical cancer.

Despite recent advances in cervical cancer diagnosis and treatment, mortality from this tumor mainly results from its metastasis to lymph nodes and distant organs [33, 34, 38]. Song et al. reported that more than 90% of patients with the earliest stage of cervical cancer survive for over 5 years after diagnosis, whereas those diagnosed at later stages (i.e., with metastasis) have poor clinical outcome [38]. These reports indicated that metastasis is the major obstacle to improving treatment and prolonging the survival of patients with cervical cancer. Moreover, several reports demonstrated that CSCs and EMT are closely correlated with metastasis [39–41]. Prompted by these reports, we further examined CSCs and EMT in cervical cancer cells to detect the role of NUSAP1 in metastasis.

CSCs have an important function in the process of metastasis in various cancers, including cervical cancer [39, 42–44]. Moreover, a growing body of evidence shows that the Wnt/β-catenin signaling correlates significantly with the CSCs self-renewal, and enhances the invasion, metastasis, and tumorigenicity [45]. However, the mechanisms underlying the maintenance of the CSC population and its clinical relevance in cervical carcinoma remain elusive. In the present study, we reported that NUSAP1 significantly enhanced tumor sphere formation and the expression levels of several stem cell-related genes in vitro, and promoted metastasis in vivo. Consistently, NUSAP1 increased the proportion of side population phenotypes cells, which is regarded as a characteristic feature of CSCs [46], indicating that NUSAP1 may promote the stemness of cervical cancer cells. Therefore, our findings revealed a new mechanism linking the maintenance of CSCs with metastasis in cervical carcinoma. Importantly, our study might also provide a potential target for reduce the population of CSCs in cervical cancer.

Numerous lines of evidence have shown that EMT has pivotal roles in metastasis in several types of cancer [15, 18, 47, 48]. In the present study, we showed that high expression of *NUSAP1* induces an EMT-like phenotypic transition and metastasis in cervical cancer cells. In addition, the Wnt/β-catenin pathway has been implicated in EMT and induces metastasis in several types of cancers [19, 49, 50]. Consistent with these reports, our study indicated that upregulation of *NUSAP1* activated Wnt/β-catenin signaling in cervical cancer cells. Moreover, inhibiting Wnt/β-catenin signaling using the tankyrase inhibitor XAV-939 and β-catenin-siRNA in *NUSAP1*-overexpressing cells confirmed that Wnt/β-catenin pathway was required for NUSAP1-mediated EMT. Although the precise mechanisms of NUSAP1-induced EMT requires further investigation, we believe that high expression of *NUSAP1* promotes EMT and induces metastasis in cervical cancer cells at least partly depending on activated Wnt/β-catenin signaling. Therefore, our data suggested that NUSAP1 might represent as a new regulator of EMT and may also represent a potential therapeutic target in cervical cancer.

Previous reports showed that Wnt/β-catenin signaling promotes invasion, metastasis, and tumorigenesis [51–54]. Nevertheless, the precise mechanism that maintains stemness and promotes EMT via the Wnt/β-catenin pathway in cervical cancer remains unclear. In this study, we identified that NUSAP1 interacted with an nuclear pore complex (NPC) protein, RanBP2 and TCF4. We also found that TCF4 was SUMOylation by RanBP2. Modification of proteins by SUMO is involved in a large variety of molecular processes such as protein-protein interaction, subcellular localization and transcriptional activity [55]. Our results also reveal that NUSAP1 increased the nuclear import of the TCF4 and β-catenin and their transcriptional activity via interacting with RanBP2 protein, and then enhanced CSCs properties and EMT processes in cervical cancer. SUMO1 modification appears to be an“ON/OFF”switch that regulates the nuclear import of β-catenin [56]. The final output of Wnt signaling is the formation of TCF/LEF and β-catenin nuclear complexes. NUSAP1 overexpression induced SUMOylation of TCF4 via binding NPC protein, RanBP2, is involved in the process of cervical cancer metastasis and progression. Wnt/β-catenin signaling has been indicated in manymalignancies, targeting this pathway could represent an attractive anticancer therapy. In the present study, a small molecule, XAV-939, a WNT signaling inhibitor, was used to inhibit catenin transcription [57]. Notably, adding XAV-939 remarkably repressed cancer stem cell properties and metastasis processes in cervical cancer cells. Metastasis is the main obstacle affecting the survival of patients with advanced cervical cancer, thus, identifying biomarkers and genotypes for predicting metastasis in the progression of cervical cancer and preventing the development of metastasis will be beneficial for a large group of cervical cancer patients. The results of these studies will not only contribute to our understanding of NUSAP1 to cervical cancer metastasis but will also lead to the characterization of specific tumor patients’ groups with NUSAP1 overexpression who may benefit from co-therapy of traditional treatment and XAV-939.

## Conclusions

In summary, NUSAP1 contributes to metastasis of cervical cancer by inducing the sumoylation of TCF4 via interact with SUMO-E3 ligase Ran-binding protein2 (RanBP2) and then enhance the Wnt/β-catenin signaling activity. Moreover, NUSAP1 could be used to predict postoperative metastasis and recurrence in patients with cervical cancer, and a Wnt/β-catenin pathway inhibitorXAV-939 may serve as a potential tailored treatment for NUSAP1-ovexpressed cervical cancer patients. Moreover, future studies are needed to determine the in-depth mechanisms underlying metastasis induced by upregulation of NUSAP1.

## Additional files

Additional file 1: **Table S1:** Clinicopathological characteristics and tumor expression of NUSAP1 in cervical cancer patients. (DOCX 17 kb)

Additional file 2: **Table S2.** Cox regression univariate andmultivariate analyses of prognostic factors in cervical cancer patients. (DOCX 15 kb)

Additional file 3: **Figure S4.** (A-G). GSEA plot showing that NUSAP1 expression positively correlated with metastasis in GSE 3325 datasets. (TIF 909 kb)

Additional file 4: **Figure S5.** (A-E). GSEA plot showing that NUSAP1 expression positively correlated with metastasis and cancer stem cell in GSE 6919 datasets. (TIF 1119 kb)

Additional file 5: **Figure S1.** (A). Western blot analysis of NUSAP1 expression in the indicated cells. GAPDH was used as a loading control. (TIF 266 kb)

Additional file 6: **Figure S2.** (A-D). Stable overexpress or silence NUSAP1 in Hela and Siha cell lines. Cells were assessed for proliferation by MTT assays. Values are the mean ± SD of three independent experiments. *P*-values were calculated using the two-tailed Student’s t-test. (E-G). Xenograft model in nude mice. (E). Representative graph of tumor growth five weeks after inoculation. (F-G). Tumor vulumes were measured on the indicated days. All data are shown as mean ± SD, *P*-values were calculated using the two-tailed Student’s t-test. (TIF 847 kb)

Additional file 7: **Figure S3.** (A, B). Real-time PCR analysis of the mRNA expression levels of the candidate downstream targets of Wnt/β-catenin in the indicated cells. (TIF 121 kb)

## Abbreviations

ATCC: American type culture collection
BEGF: Basic fibroblast growth factor
BMI1: B lymphoma Mo-MLV insertion region 1 homolog
CSC: Cancer stem cell
DAPI: 4,6 diamidino-2-phenylindole
DMEM: Dulbecco’s modified eagle medium
EGF: Epidermal growth factor
EMEM: Eagle’s Minimum Essential Medium
EMT: Epithelial-mesenchyme transition
FBS: Fetal bovine serum
GAPDH: Glyceraldehyde-3-phosphate dehydrogenase
GEO: The Gene Expression Omnibus
GSEA: Gene set enrichment analysis
H&E: Hematoxylin-eosin
LRP5/6: Lipoprovein receptor-related protein-5 or − 6
MMP7: Matrix metalloproteinase 7
MMP9: Matrix metalloproteinase 9
NANOG: homeobox transcription factor Nanog
NPC: Nuclear pore complex
NUSAP1: Nucleolar and spindle associated protein 1
OCT4: Octamer-binding protein 4
PBS: Phosphate-buffered saline
qRT-PCR: Quantitative real-time reverse transcription polymerase chain reaction
RanBP2: Ran binding protein-2
RPMI-1640: Roswell Park Memorial Institute-1640
SD: Standard deviation
SI: The staining intensity
SOX2: SRY-Box 2
SUMO1: The small ubiquitin-related modifier
TCF/LEF: T-cell factor/lymphoid enhancer factor
TCF4: T-cell factor-4
TCGA: The Cancer Genome Altlas
