# Supplementary material for: Nucleolar and spindle associated protein 1 promotes metastasis of cervical carcinoma cells by activating Wnt/β-catenin signaling
Source: J Exp Clin Cancer Res. 2019 Jan 24;38:33. doi: 10.1186/s13046-019-1037-y (PMC6346521; doi:10.1186/s13046-019-1037-y)
Supplement: Supplementary file 1 — Table S1: Clinicopathological characteristics and tumor expression of NUSAP1 in cervical cancer patients. (DOCX 17 kb) [file 13046_2019_1037_MOESM1_ESM.docx]

**Table 1.** Clinicopathological characteristics and tumor expression of NUSAP1 in cervical cancer patients.

| **Characteristic** | **Number of cases (%)** |
| --- | --- |
| **Age (years)** |  |
| ≤ 43 | 121(51.9) |
| > 43 | 112 (48.1) |
| **FIGO stage**  I  II  III | 140(60.3)  90( 38.8)  2(0.9) |
| **Tumor size, cm** |  |
| < 4 | 201(86.3) |
| ≥ 4 | 32 (13.7) |
| **Squamous cell carcinoma antigen (ng/ml)** |  |
| ≤ 1.5 | 135 (63.7) |
| > 1.5 | 77 (36.3) |
| **Pelvic lymph node metastasis** |  |
| No | 178(76.4) |
| Yes | 55 (23.6) |
| **Tumor recurrence** |  |
| No | 207(88.8) |
| Yes | 26(11.2) |
| **Vital status (at last follow-up)** |  |
| Alive | 151(64.8) |
| Dead | 82 (35.2) |
| **Differentiation grade** |  |
| G1 | 17 (7.7) |
| G2 | 65(29.5) |
| G3 | 138(62.8) |
| **Deep Myometrium invasion** |  |
| No | 108(46.4) |
| Yes | 125(53.6) |
| **Property of surgical margin** |  |
| No | 221(95.7) |
| Yes | 10 (4.3) |
| **Infiltration of parauterine organ** |  |
| No | 226(97.0) |
| Yes | 7 (3.0) |
| **Lymphovascular space involvement** |  |
| No | 205(88.0) |
| Yes | 28(12.0) |
| **Concurrent Chemotherapy and radiotherapy** |  |
| No | 166(71.2) |
| Yes | 67(28.8) |
|  |  |
| **Expression of NUSAP1 protein** |  |
| Low or none | 125 (53.6) |
| High  **HPV Infection**  No  Yes  **Chemotherapy**  No  Yes | 108(46.4)  91(63.2)  53( 36.8)  108(46.4 )  125(53.6 ) |
