# Supplementary material for: Nucleolar and spindle associated protein 1 promotes metastasis of cervical carcinoma cells by activating Wnt/β-catenin signaling
Source: J Exp Clin Cancer Res. 2019 Jan 24;38:33. doi: 10.1186/s13046-019-1037-y (PMC6346521; doi:10.1186/s13046-019-1037-y)
Supplement: Supplementary file 2 — Table S2. Cox regression univariate andmultivariate analyses of prognostic factors in cervical cancer patients. (DOCX 15 kb) [file 13046_2019_1037_MOESM2_ESM.docx]

**Supplemental table 2**

**Univariate Analysis**

**Variable Patients (n)  *P* Hazard ratio (95%CI)**

**NUSAP1**

Low expression 125 <0.001 2.250（1.434-3.529）

High expression 108

**LN status**

Without metastasis 178 <0.001 3.795（2.448-5.885）

Metastasis 55

**Deep Myometrium**

**Invasion**

No 108 <0.001 2.339（1.460-3.746）

Yes 125

**Post-operative therapy**

No 166 0.002 2.027（1.305-3.148）

Yes 67

**tumor size**

0.233 0.900（0.758-1.070）

**Stage (FIGO)**

I 140 0.241 1.286（0.845-1.959）

II 90

III 2

**SCC antigen**

0.926 1.006（0.890-1.137）

**Age**

0.407 1.009（0.988-1.031）

**Smoking status**

No 233

**Multivariable analysis**

**Variable Patients (n)  *P* Hazard ratio (95%CI)**

**NUSAP1**

Low expression 125 <0.001 2.315 (1.473-3.640)

High expression 108

**LN status**

Without metastasis 178 <0.001 3.214 (1.922-5.374)

Metastasis 55

**Deep Myometrium**

**Invasion**

No 108 0.397 1.275 (0.727-2.237)

Yes 125

**Post-operative therapy**

No 166 0.434 1.222 (0.740-2.017)

Yes 67
