# Supplementary figures and images for: Nucleolar and spindle associated protein 1 promotes metastasis of cervical carcinoma cells by activating Wnt/β-catenin signaling
Source: J Exp Clin Cancer Res. 2019 Jan 24;38:33. doi: 10.1186/s13046-019-1037-y (PMC6346521; doi:10.1186/s13046-019-1037-y)

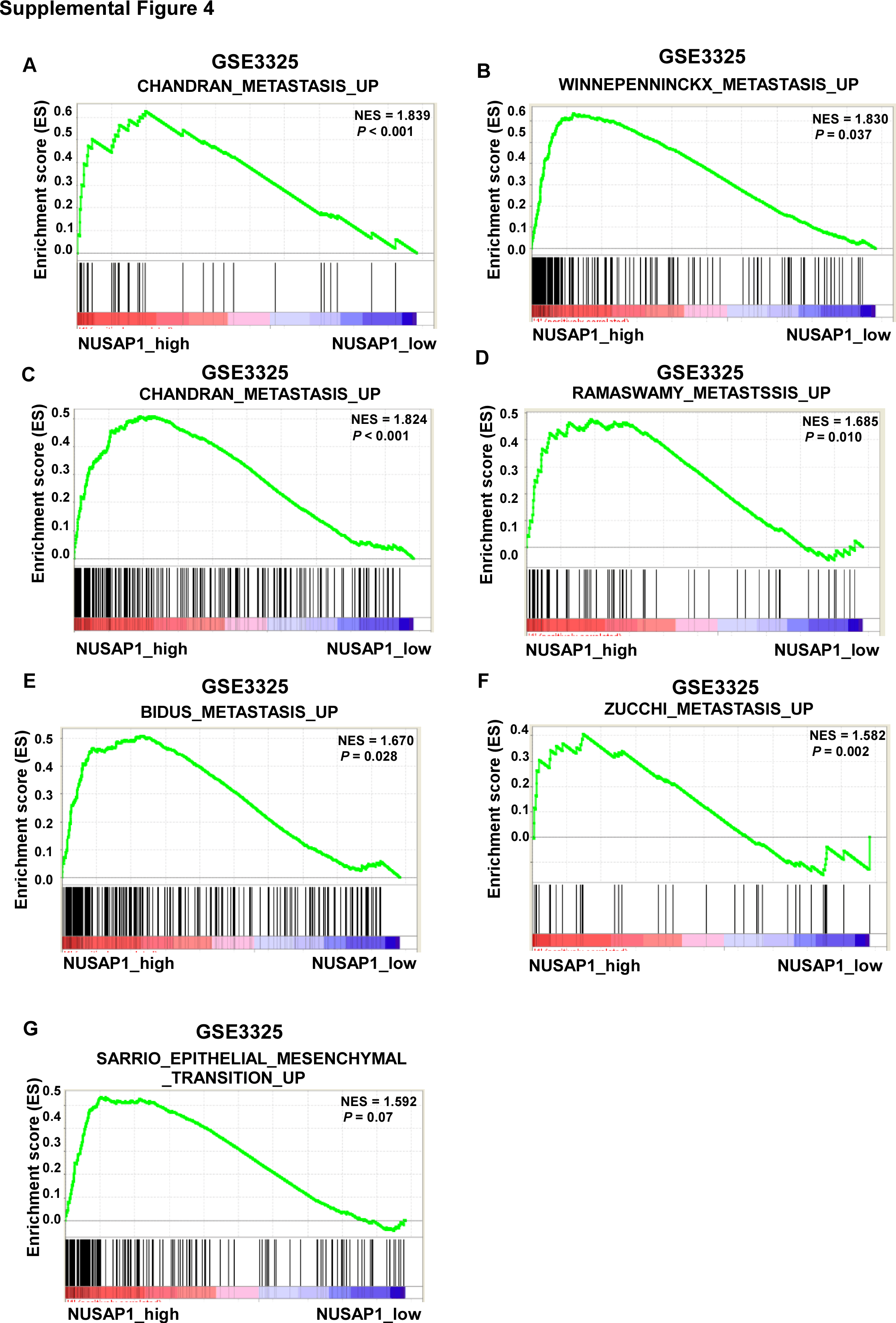

Supplement: Supplementary file 3 — Figure S4. (A-G). GSEA plot showing that NUSAP1 expression positively correlated with metastasis in GSE 3325 datasets. (TIF 909 kb) [file 13046_2019_1037_MOESM3_ESM.tif]

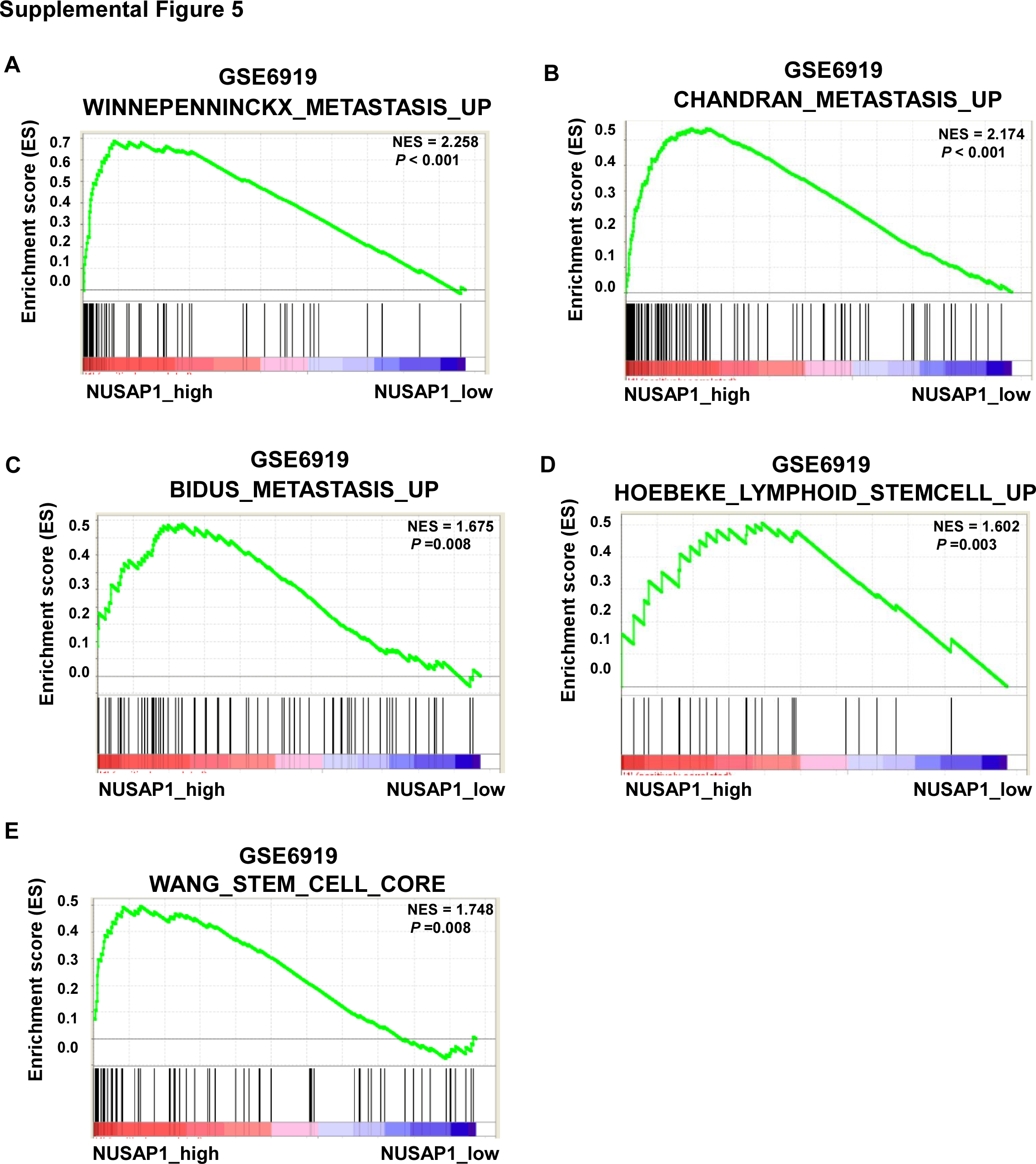

Supplement: Supplementary file 4 — Figure S5. (A-E). GSEA plot showing that NUSAP1 expression positively correlated with metastasis and cancer stem cell in GSE 6919 datasets. (TIF 1119 kb) [file 13046_2019_1037_MOESM4_ESM.tif]

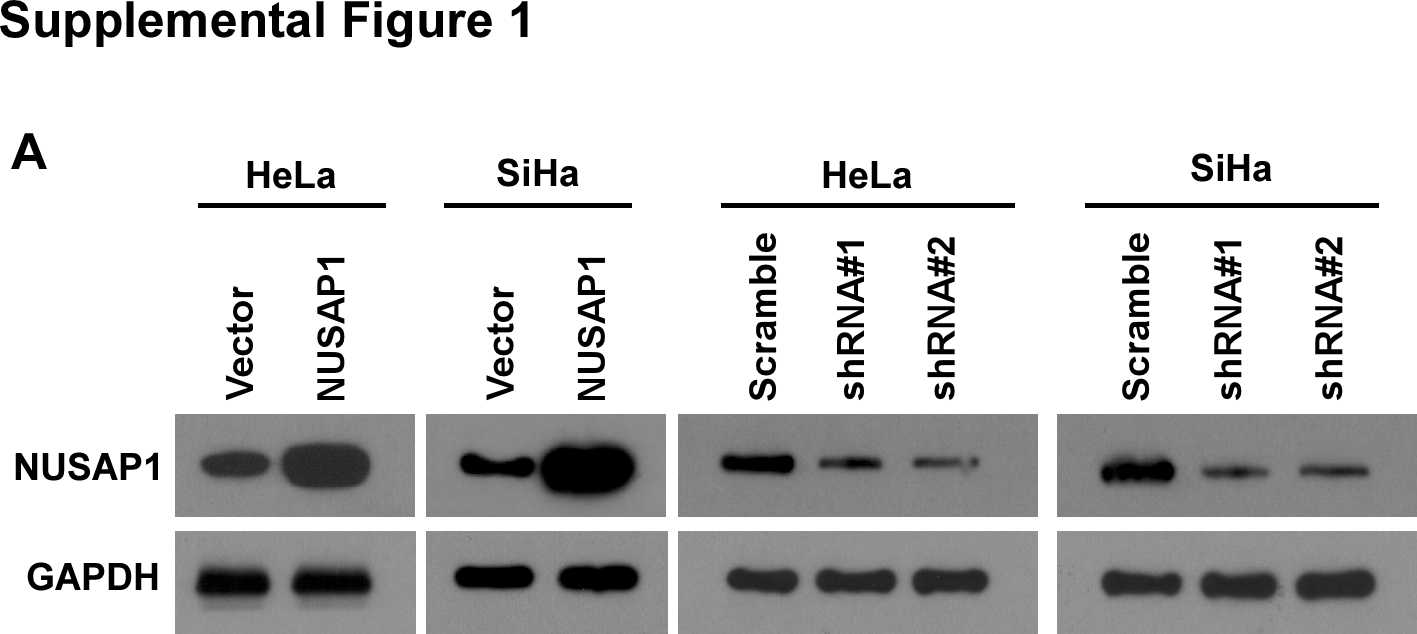

Supplement: Supplementary file 5 — Figure S1. (A). Western blot analysis of NUSAP1 expression in the indicated cells. GAPDH was used as a loading control. (TIF 266 kb) [file 13046_2019_1037_MOESM5_ESM.tif]

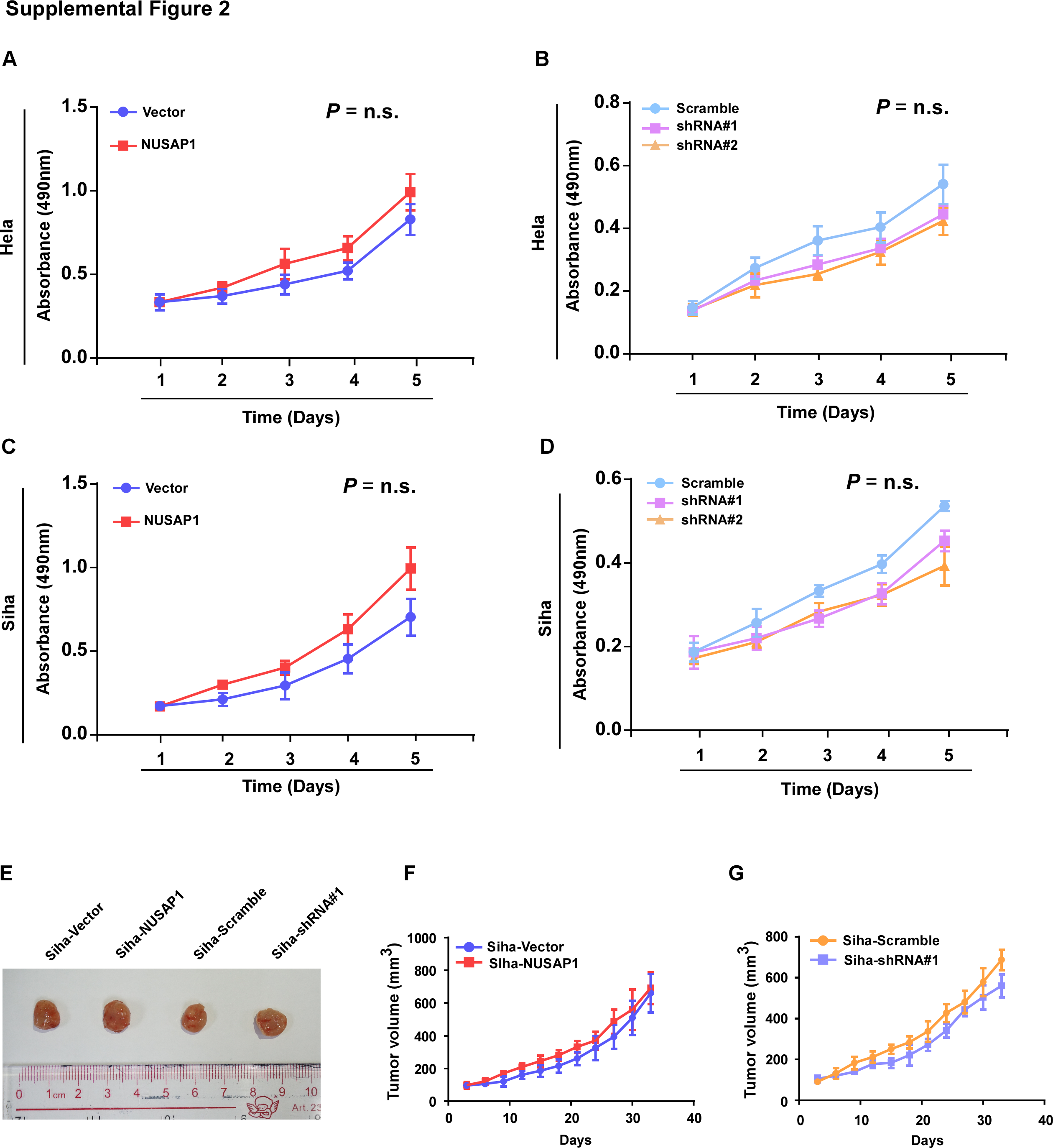

Supplement: Supplementary file 6 — Figure S2. (A-D). Stable overexpress or silence NUSAP1 in Hela and Siha cell lines. Cells were assessed for proliferation by MTT assays. Values are the mean ± SD of three independent experiments. P-values were calculated using the two-tailed Student’s t-test. (E-G). Xenograft model in nude mice. (E). Representative graph of tumor growth five weeks after inoculation. (F-G). Tumor vulumes were measured on the indicated days. All data are shown as mean ± SD, P-values were calculated using the two-tailed Student’s t-test. (TIF 847 kb) [file 13046_2019_1037_MOESM6_ESM.tif]

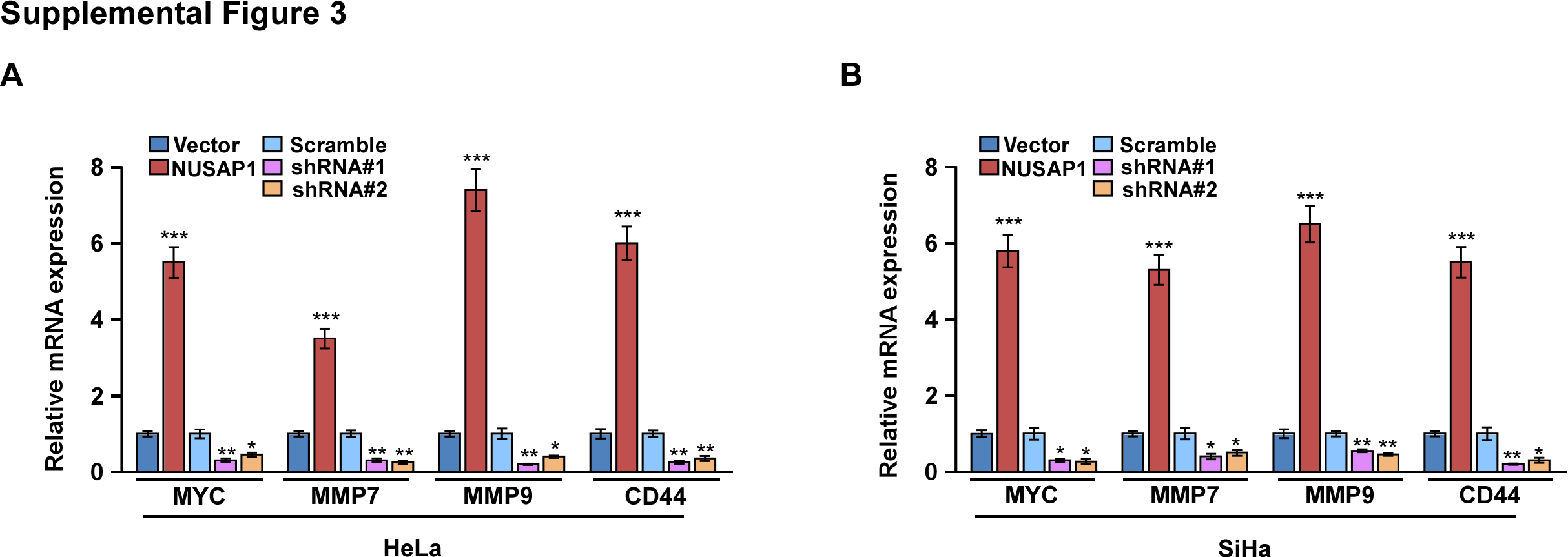

Supplement: Supplementary file 7 — Figure S3. (A, B). Real-time PCR analysis of the mRNA expression levels of the candidate downstream targets of Wnt/β-catenin in the indicated cells. (TIF 121 kb) [file 13046_2019_1037_MOESM7_ESM.tif]
